# 83 Screen & Intervene : Mental Health Screening in the Burn Clinic

**DOI:** 10.1093/jbcr/irad045.057

**Published:** 2023-05-15

**Authors:** Linda Colarusso, Eli Strait, Jeanne Lee, Alan Smith

**Affiliations:** UC San Diego Medical Center, San Diego, California; UC San Diego Health, San Diego, California; UC San Diego Medical Center, San Diego, California; UC San Diego, San Diego, California; UC San Diego Medical Center, San Diego, California; UC San Diego Health, San Diego, California; UC San Diego Medical Center, San Diego, California; UC San Diego, San Diego, California; UC San Diego Medical Center, San Diego, California; UC San Diego Health, San Diego, California; UC San Diego Medical Center, San Diego, California; UC San Diego, San Diego, California; UC San Diego Medical Center, San Diego, California; UC San Diego Health, San Diego, California; UC San Diego Medical Center, San Diego, California; UC San Diego, San Diego, California

## Abstract

**Introduction:**

Studies have demonstrated an increased prevalence of depression and PTSD in individuals with burn injuries during the first year of recovery. Mental health screening of patients at all burn centers is recommended. With the addition of an outpatient social worker, our burn center identified a need to standardize the burn clinic’s mental health screening process to address the mental health needs of patients. This performance improvement project sought to improve mental health screening in the burn clinic and subsequent referrals to community resources.

**Methods:**

From March to August of 2022, the PHQ-2 and PC-PTSD were administered to 99 clinic patients during one of the patient’s initial visits. Patients completed the screening independently using a paper form. For pediatric patients < 12 years old, the primary caregiver filled out the form. Of the 99 patients who completed the screening, 51 were previously admitted to our inpatient burn center and 48 were treated only as outpatients (F=36, M=63). The age range of patients was 10 months to 88 years old. TBSA % burned ranged from .15% to 70%. A score of 2 or more on either of the screening tools resulted in further assessment by the social worker to determine appropriate next steps. The chi-square test was used to analyze the data collected.

**Results:**

A total of 36/99 patients screened positive for depressive symptoms, 33/99 screened positive for Acute Stress Disorder (ASD)/Post Traumatic Stress Disorder (PTSD) symptoms, and 23/99 screened positive for both depressive and ASD/PTSD symptoms. All patients with a positive screen on either tool were provided with education regarding mental health resources and offered a referral for services. Of the 99 patients screened, 26 accepted a referral to the local burn foundation and/or an alternate mental health provider for support services. The other 73/99 declined a referral for follow up. There were statistically significant correlations between positive ASD/PTSD screenings and inpatient hospitalization (22/99 *p=0.033*), PTSD and median TBSA *(p= 0.011*), and PTSD and age (45-64yo, *p=0.028*). Females (19/36, *p=0.01*) were more likely than males to report symptoms of depression and both PTSD & depression *(p=0.001)*, while females (12/36) and males (21/63) were equally likely to report symptoms of PTSD.

**Conclusions:**

Mental health screening of individuals in our burn clinic yielded a significant number of positive screens for depression and ASD/PTSD symptoms, validating the need for a standardized screening process. Positive screens resulted in increased referrals to community and mental health resources.

**Applicability of Research to Practice:**

Performing mental health screenings in the outpatient arena is vital to address the mental health needs of individuals with burn injuries.

